# Management of Cataract in Patients with Age-Related Macular Degeneration

**DOI:** 10.3390/jcm10122538

**Published:** 2021-06-08

**Authors:** Hemal Mehta

**Affiliations:** 1 Save Sight Registries, University of Sydney, Sydney, NSW 2000, Australia; HM@cantab.net; 2 Strathfield Retina Clinic, Strathfield, Sydney, NSW 2135, Australia; 3 Ophthalmology Department, Royal Free London NHS Foundation Trust, London NW3 2QG, UK

**Keywords:** cataract surgery, age-related macular degeneration, clinical trials, real-world evidence

## Abstract

Cataract and age-related macular degeneration (AMD) are two of the most common eye diseases of aging. This review addresses the pre-operative, intra-operative, and post-operative considerations in managing cataract in patients with age-related macular degeneration. Surgery for visually significant cataracts in patients with AMD can substantially improve the quality of life and reduce the risk of falls. Pre-operative optical coherence tomography is now recommended where possible to identify pre-existing macula disease. Careful counselling of patients is required before cataract surgery, especially with respect to the expected visual outcome, intraocular lens choice and potential risks of surgery. Real-world data has suggested 6 months of intravitreal anti-VEGF therapy for neovascular AMD before cataract surgery is compatible with optimum long-term visual outcomes. Patients receiving intravitreal therapy for neovascular AMD should be advised of the slightly higher risk of intraoperative complications and the surgeon should be prepared to manage these during the operation. During cataract surgery, unnecessary light exposure should be avoided to reduce phototoxicity. Careful planning of intravitreal therapy for neovascular AMD just before cataract surgery allows the eye greater recovery time in the post-operative period before further planned intravitreal therapy.

## 1. Introduction

Cataract and age-related macular degeneration (AMD) are two of the most common causes of visual impairment globally, with the incidence set to increase in upcoming decades with an ageing population [1]. A recent study in patients over 50 years of age identified that 20% of 411 eyes listed for cataract surgery had some evidence of AMD on optical coherence tomography (OCT) imaging [2]. This review provides guidance on the pre-operative, intra-operative, and post-operative considerations in managing cataracts in patients with age-related macular degeneration (Table 1). Evidence is derived from available clinical trials, real-world evidence and expert opinion.

## 2. Pre-Operative Considerations

### 2.1. Counselling Regarding Visual Acuity and Quality of Life Outcomes

The Age-Related Eye Disease Studies (AREDS1 and AREDS2), identified a mean improvement of +4 and +11 logMAR letters in those with intermediate AMD undergoing cataract surgery [3,4]. Additionally, for patients with driving level vision (better than 20/40 Snellen), final visual acuity (VA) outcomes were similar to control patients.

In neovascular AMD, a post-hoc analysis of the MARINA (Minimally Classic/Occult Trial of the anti-VEGF Antibody Ranibizumab in the Treatment of Neovascular AMD) and ANCHOR (Anti-VEGF Antibody for the Treatment of Predominantly Classic Choroidal Neovascularization in AMD) clinical trials demonstrated that cataract surgery was beneficial for eyes with an average improvement of >10 logMAR letters [5]. 

Although cataract surgery may not reliably improve VA in fovea-involving geographic atrophy, it may improve other critical aspects of visual function such as contrast sensitivity, peripheral vision, glare, and color vision as well as help with quality of life indices [6,7,8].

A recent study identified the risk of falls decreased by 54% (incidence rate ratio (IRR) = 0.46, 95% CI = 0.22–0.97, *p* = 0.04) after first eye cataract surgery only, compared with the period before the first eye surgery [9]. The risk of falls decreased by 73% (IRR = 0.27, 95% CI = 0.11–0.63, *p* = 0.002) after the second eye cataract surgery, compared with the period before the first eye surgery. Improved binocular VA (IRR = 5.49, 95% CI = 1.19–25.28, *p* = 0.03) and contrast sensitivity (IRR = 0.26, 95% CI = 0.070–0.94, *p* = 0.04) were associated with a decrease in the number of falls.

### 2.2. Does Cataract Surgery Cause AMD to Progress?

There has been much debate and conflicting evidence as to whether cataract surgery causes AMD progression [10]. A Cochrane review of two small randomised controlled trials recommended that physicians must make recommendations to their patients with AMD regarding cataract surgery based on experience and clinical judgment until large controlled trials are conducted and their findings published [11,12,13]. It concluded that although cataract surgery provides improvement in vision in eyes with AMD compared with no surgery at six months, it is unclear whether the timing of surgery has an effect on longer-term visual outcomes. The Cochrane review also stated that “*ethical considerations preclude withholding surgery, or delaying it for several years, if it may be a potentially beneficial treatment*” [11]. Another systematic review that included a variety of study designs concluded the link between cataract surgery and AMD remained equivocal due to limited available evidence [14].

### 2.3. Screening for Macula Disease with Optical Coherence Tomography

A number of studies have identified the benefit in screening eyes with macula OCT imaging prior to cataract surgery [2,15,16]. In one study, all patients underwent routine spectral-domain OCT scanning prior to cataract surgery [2]. The scans were reviewed by a retinal specialist for macular pathology and compared to pre-operative biomicroscopic fundus examination findings. Overall, the management of 107 (26.0%) out of 411 patients was modified due to macular spectral-domain OCT findings, which were either missed (22.8%) or underestimated (3.2%) by the fundus biomicroscopy examination. Changes in pre-operative patient management included altering patient consultation regarding presbyopia correction options (73 eyes, 17.8%) and referral to a retina specialist (34 eyes, 8.3%). Routine macular spectral-domain OCT scans for cataract surgery candidates helped to identify macular pathologies that might be missed or underestimated by standard fundus biomicroscopy examination. 

### 2.4. How Long Does Neovascular AMD Need to Be Treated with Intravitreal Therapy before Considering Cataract Surgery?

The Fight Retinal Blindness (FRB!) registry investigated the timing of cataract surgery in patients with neovascular AMD [17]. Patients who had cataract surgery within 6 months of initiating anti-VEGF therapy were more likely to lose vision. It is possible that only after the first 6 months after commencing anti-VEGF therapy the lesions were sufficiently controlled. The average time from the first injection to cataract surgery was 14 months in the post hoc analysis of the ANCHOR and MARINA clinical trials [5]. A preoperative exudation-free period may be an important parameter when considering cataract surgery in patients with neovascular AMD, although the exact minimum period of inactivation has not yet been determined [18]. Cataract surgery within 6 months of starting treatment for neovascular AMD should be avoided if possible. 

### 2.5. Predicting Visual Acuity Outcomes

Predicting the extent of VA improvement in patients with concurrent cataract and AMD is challenging. The FRB! registry identified that the angiographic subtype of neovascular AMD did not impact the visual outcomes of cataract surgery [17]. Prognostic factors in patients with AMD undergoing cataract surgery have recently been investigated in a retrospective study [19]. Better pre-operative VA predicted smaller VA gains (*p* < 0.007). Longer duration of AMD in intermediate AMD, ellipsoid zone disruption in neovascular AMD, and lower central subfield thickness in geographic atrophy were associated with poorer VA outcomes (*p* < 0.05). Further studies are required to quantify the impact of these biomarkers on visual outcomes to allow for more accurate counselling of patients pre-operatively.

### 2.6. Discussion of Intraocular Lens Choices with Patients

There are a number of specific considerations of intraocular lens (IOL) choice in patients with AMD undergoing cataract surgery:

#### 2.6.1. Avoiding Multifocal Intraocular Lenses

Multifocal IOLs aim to address presbyopia by splitting light rays to different focal points to reduce spectacle dependence [20]. They are classified as refractive, diffractive, or a combination of these [20]. Bifocal and now trifocal IOL options are available. Multifocal IOLs are however associated with reduced contrast sensitivity and more higher order aberrations than monofocal IOLs [20]. Multifocal IOLs are relatively contraindicated in patients with AMD [21].

There is a suggestion that extended depth of focus (EDoF) IOLs may have less of a deleterious effect on contrast sensitivity and cause less higher order aberrations than trifocal IOLs [22]. However, the quality of vision would still be expected to be worse than a monofocal IOL [23]. Evidence that EDoF IOLs are well tolerated in patients in AMD would be required before advocating them.

#### 2.6.2. Toric Intraocular Lenses 

Toric IOLs do not compromise contrast sensitivity and can be used in patients with AMD if there is significant astigmatism [24]. Toric IOLs do need to be dialed to a specific alignment. Therefore, a back-up non-toric IOL should be available in case of zonular weakness. 

#### 2.6.3. Aspheric Intraocular Lenses

Aspheric IOLs negate the positive spherical aberrations of the cornea. Clinical trials have reported improved contrast sensitivity, glare sensitivity and coma compared with conventional IOLs [25,26,27]. This could be beneficial in patients with AMD although at this stage further evidence in this patient population is required to support this hypothesis [28].

#### 2.6.4. Blue-Blocking Intraocular Lenses

Ultraviolet filtering IOLs that block harmful UV-C energy outside the visible spectrum reaching the macula have been available for over 30 years [29]. Most commercially available intraocular lenses contain this filter as a default and there is widespread acceptance of the benefit in the medical community. 

The story with blue light filtering IOLs is less clear cut [30]. Blue light–blocking IOLs are designed to filter short-wavelength light in the visible spectrum in addition to ultraviolet light and mimic the natural crystalline lens. An in vitro study identified that an ultraviolet- and blue light-absorbing intraocular lens demonstrated significantly better protection against light-induced oxidative stress, senescence, and structural retinal pigment epithelial damage than the ultraviolet-absorbing intraocular lens [31]. A small retrospective study reported that geographic atrophy progression in the UV-blocking IOL group to be significantly greater than the combined UV and blue-blocking IOL group after one year [32].

Conversely, blue light has a role in helping scotopic vision and suppresses melatonin, helping regulate the circadian rhythm [33,34]. Concerns about impaired night vision, disturbed sleep, and impacts on quality of vision have been raised although not definitively proven.

A registry-based cohort study with data from the Swedish National Cataract Register and the Swedish Macula Register from 2010 to 2017 compared eyes with and without preoperative AMD that had undergone cataract surgery and subsequently treated for neovascular AMD to eyes not treated within the study period [35]. All first-eye surgeries registered from 2010 to 2017 and matching eyes found in the Swedish Macula Register that had undergone treatment for neovascular AMD >1 year after the cataract procedure were included. A blue-blocking IOL did not statistically significantly decrease the likelihood of subsequent neovascular AMD treatment in eyes with pre-operative AMD (53% vs. 57%, *p* = 0.11). The authors concluded that if the use of a blue-blocking IOL offers any protection from undergoing neovascular AMD treatment after cataract surgery, such an effect must be very small.

#### 2.6.5. Intraocular Lenses Providing Magnification or Prismatic Effect

Patients with AMD can benefit from additional magnification, especially for reading tasks. Handheld magnifiers can be difficult to use especially if other comorbidities such as significant arthritis of the upper limbs is present. Some of this is mitigated by improved accessibility options on modern tablets and phones. Magnifying IOLs have been developed. The most common approach to magnification, used in implantable miniature telescope (IMT) lenses, the IOL-VIP System, and iolAMD is a Galilean type telescope, where two optical elements with high positive and negative power should be used in combination with the cornea [36]. Another approach to magnification is the Lipshitz macular implant, based on a Cassegrain configuration, which uses mirrors instead of lenses [36]. A description of all the available magnifying intraocular lens devices is outside the scope of this review but is well covered elsewhere [10,36,37]. There is an inherent compromise between improved magnification and loss of field of view. Some magnifying IOLs such as the Scharioth Macula Lens attempt to address this by having high magnification in the central optic only. Generally, magnifying IOLs are more of an option in advanced AMD. Long-term safety data is required, especially for devices that occupy significant volume in eyes with shallow anterior chambers. 

Prism-based intraocular lenses do not necessarily provide magnification but instead displace the retinal image from the damaged central macula to a more peripheral healthier area. The Fresnel prism approach has a potential problem of diffraction and scattered light at the edges of each Fresnel zone, which might be a source of significant glare [36,38].

## 3. Intra-Operative Considerations

There are factors for the surgeon to consider during cataract surgery in patients with AMD. 

### 3.1. Timing of Intravitreal Anti-VEGF Therapy and Cataract Surgery

Most modern day phacoemulsifaction cataract surgery is performed without sutures, aiding the speed of visual recovery. It is preferable to allow time for these self-sealing wounds to heal before performing further intravitreal therapy. Therefore, clinicians aim to deliver an intravitreal anti-VEGF injection for neovascular AMD in the weeks preceeding cataract surgery. In the ANCHOR and MARINA clinical trials, intravitreal ranibizuamb was delivered in the month prior to cataract surgery with good outcomes [5]. 

There have also been studies reporting good outcomes when delivering intravitreal anti-VEGF at the time of cataract surgery to reduce the risk of activating the choroidal neovascular (CNV) lesion [39,40,41]. However, the FRB! registry did not observe a relationship between intravitreal anti-VEGF for neovascular AMD in the two weeks before the cataract surgery and VA outcomes [17]. This is in contrast to diabetic macular oedema, where the FRB! registry identified benefit of intravitreal therapy even if at the time of cataract surgery [42]. 

If intravitreal anti-VEGF therapy is anticipated in the first week after cataract surgery, it may be prudent to place a suture to secure the main wound.

### 3.2. Managing Posterior Capsular Rupture or Zonular Dialysis during Cataract Surgery

Eyes receiving intravitreal therapy for neovascular AMD appear to be at increased risk of intra-operative complications during cataract surgery. Possible explanations include inadvertent crystalline lens capsule trauma and zonular trauma either directly or from local scleral deformation at the time of intravitreal injections [43]. Identification of cases at higher risk assists the operative planning and allows patients to be better informed about potential surgical risks.

Posterior capsular rupture (PCR) is a complication of cataract surgery that can be associated with significantly worse visual outcomes [44]. The Royal College of Ophthalmologists of England National Ophthalmology Database study of cataract surgery for cases between 2010 and 2018 reported a mean PCR rate of just less than 1% [45]. The hypothesis that previous intravitreal therapy is a predictor of increased risk of PCR during cataract surgery was tested on a large registry of eyes undergoing cataract surgery from 20 UK Hospital Trusts between 2004 and 2014 [46]. Data were available on 65,836 cataract operations, of which 1935 eyes had received previous intravitreal injections (2.9%). Of these injections, 80% were intravitreal anti-VEGF therapy for neovascular AMD. Univariate regression identified advanced cataract, patient age, junior cataract surgeon grade, and the number of previous intravitreal injections were associated with increased risk of PCR. Analysis considering intravitreal injections as a categorical variable identified 10 or more previous injections that were associated with a 2.6 times greater likelihood of PCR (*p* = 0.003) after adjusting for other significant independent predictors. Three independent studies have supported this observation, one from the USA, and two others from the UK [47,48,49]. Much like we see with COVID-19 vaccine development, rare adverse events can be identified outside of a clinical trial setting in real-world settings [50].

Cataract surgeons may have to manage zonular dialysis in patients who have received previous intravitreal anti-VEGF therapy. This seems biologically plausible given the proximity of zonules to the site of intravitreal injections, although at this stage there is no supportive evidence. A large retrospective study identified improved short and medium-term visual outcomes when a capsule tension ring (CTR) was used for zonule dialysis [28]. The records of 22,312 consecutive eyes undergoing cataract surgery were reviewed. The incidence of zonular dialysis was 0.50% (111 eyes). A CTR was inserted in 46 eyes. Using a multivariate linear regression model, better initial pre-operative VA (*p* = 0.019), the use of a CTR (*p* = 0.014), and the absence of vitreous loss during surgery (*p* = 0.008) were associated with improved early postoperative VA. Better medium-term postoperative VA was significantly associated with the use of a CTR during surgery (*p* = 0.004). 

### 3.3. Reducing Unnecessary Light Exposure

Phototoxicity to the retina has been described with solar retinopathy or laser-light induced retinopathy [51,52]. The macula is already not healthy in AMD and hence may be more vulnerable to phototoxic damage [53]. Prolonged surgery and bright light settings during cataract surgery are potential risk factors for phototoxic macula damage [53]. A balance has to be struck between reducing light exposure and adequate visualization to perform the surgery safely. The visually significant cataract should reduce the extent of light exposure early in the cataract operation. Once the new intraocular lens is inserted, UV light should be blocked. The main time of risk would therefore be when the eye is aphakic and the surgeon can take extra precautions to reduce light exposure during this period of the cataract surgery. 

### 3.4. Femtosecond Laser-Assisted Cataract Surgery

A small retrospective study compared femtosecond laser and conventional cataract surgery for patients with neovascular AMD undergoing cataract surgery [54]. Overall, the postoperative course between neovascular AMD after femtosecond laser and conventional cataract surgery was equal. During the early follow-up, however, femtosecond laser-treated eyes had less subclinical macular oedema on optical coherence tomography imaging, suggesting that the role of femtosecond laser-assisted cataract surgery in eyes with macular vulnerability is an area for further research. It may be that the shorter phacoemulsification time reported in femtosecond laser-assisted cataract surgery leads to less cystoid macular oedema.

## 4. Post-Operative Considerations

### 4.1. Increased Risk of Acute and Delayed Endophthalmitis

Over 200,000 Medicare beneficiaries in the USA underwent cataract surgery from 1 January 2009 to 31 December 2013. By using a 5% sample of Medicare claims data, the risks of adverse outcomes in beneficiaries with a history of intravitreal injections relative to those without were calculated using the Cox proportional hazard model [55]. Prior injections were associated with increased risk of both acute (HR, 2.29; 95% CI, 1.001–5.22) and delayed-onset endophthalmitis (HR, 3.65; 95% CI, 1.65–8.05). The authors recommended increased post-operative vigilance in patients with a history of intravitreal injections undergoing cataract surgery.

### 4.2. Intravitreal Therapy for Patients with Neovascular AMD after Cataract Surgery

The FRB! registry investigated whether there was a change in the frequency of intravitreal anti-VEGF injections required for neovascular AMD in the 12 months after cataract surgery compared with the 12 months prior [17]. Disease activity grading and intravitreal injection numbers were similar in both periods for patients having cataract surgery, whereas both slightly decreased in the control group in the latter 12 months, suggesting that cataract surgery modestly increased the level of activity of the CNV lesion. Studies without a control group suggested cataract surgery did not influence CNV activity [56,57].

### 4.3. Consider Additional Lighting, Magnification and Reading Aids

After cataract surgery, the benefit of additional lighting or magnification in patients with AMD should be considered. Patients may find low vision aids more beneficial once they have clear media after cataract surgery. Local patient support groups can offer routes to access these. Modern portable electronic devices have desirable properties such as the flexible use of magnification. 

Glasses are usually updated one month after cataract surgery. There was no good evidence from a Cochrane Review of low vision aids to support the use of filters or prism spectacles in patients with low vision [58].

**Table 1:** Summary of factors to consider when carrying out cataract surgery in patients with age-related macular degeneration.

	Recommendation/Consideration	Level of Evidence
**Pre-operative**	There are quality of life benefits in carrying out surgery for visually significant cataract in patients with all stages of AMD	Level 2
	Screening for macula disease with pre-operative optical coherence tomography is recommended	Level 2
	Avoid multifocal intraocular lenses in patients with macular disease	Level 3
	Cataract surgery within 6 months of starting treatment for neovascular AMD should be avoided if possible.	Level 2
	Intravitreal anti-VEGF therapy for neovascular AMD in the month before cataract surgery is compatible with good long-term visual outcomes.	Level 2
**Intra-operative**	Slightly increased risk of posterior capsule rupture in eyes that have received intravitreal therapy	Level 2
	Reduce unnecessary light exposure during cataract surgery	Level 3
**Post-operative**	Slightly increased risk of acute or delayed endophthalmitis in patients undergoing cataract surgery who have had previous intravitreal injections.	Level 2
	The frequency of intravitreal anti-VEGF therapy for eyes with neovascular AMD is likely to be similar in the 12 months before and after cataract surgery	Level 2
	Offer access to additional lighting, magnification and reading aids	Level 2

Evidence is graded on three levels [59]: Level 1: evidence based on results of randomised controlled trials, power calculations, or other recognised means to determine the statistical validity of the conclusion. There is a lack of high-quality Level 1 evidence in the field. Level 2: evidence based on results of case studies, case series, or other non-randomised prospective or retrospective analysis of patient data. Level 3: evidence based on expert opinion, consensus opinion, or current recognised standard of care criteria where no formalcase series analysis was available.

## 5. Conclusions

Real-world evidence has complemented the limited available clinical trial data to provide useful insights into how to optimize outcomes in patients with AMD undergoing cataract surgery. Further studies are required to provide more accurate prediction of the visual potential of eyes undergoing cataract surgery, to understand whether blue-blocking intraocular lenses offer long-term visual benefits, to investigate the long-term safety of magnifying intraocular lenses, and to identify optimal surgical techniques. It may be that some of these questions can be answered by clinical trials whereas others will be better addressed by large registry studies.
